# Efficacy and Safety of Non-Surgical Treatments for Pancreatic Neuroendocrine Tumors: A Systematic Review and Meta-Analysis

**DOI:** 10.3390/ph18111650

**Published:** 2025-10-31

**Authors:** Mohammed Saad AlQahtani, Bogdan Miutescu, Ielmina Domilescu, Serban Negru, Dorel Popovici, Eyad Gadour

**Affiliations:** 1 Liver Transplantation Unit, Multiorgan Transplant Centre of Excellence, King Fahad Specialist Hospital, Dammam 32252, Saudi Arabia; alqahtanim222@gmail.com (M.S.A.); eyadgadour@doctors.org.uk (E.G.); 2 Department of Surgery, Imam Abdulrahman Bin Faisal University, Dammam 31441, Saudi Arabia; 3 Division of Gastroenterology and Hepatology, Department of Internal Medicine II, “Victor Babes” University of Medicine and Pharmacy, 300041 Timișoara, Romania; 4 Advanced Regional Research Centre in Gastroenterology and Hepatology, “Victor Babes” University of Medicine and Pharmacy, 300041 Timișoara, Romania; 5 Doctoral School, Faculty of Medicine, “Victor Babes” University of Medicine and Pharmacy, 300041 Timișoara, Romania; ielmina.domilescu@umft.ro; 6 Department of Oncology, Faculty of Medicine, “Victor Babes” University of Medicine and Pharmacy, 300041 Timișoara, Romania; serban.negru@umft.ro (S.N.); dorel.popovici@umft.ro (D.P.); 7 Department of Medicine, Faculty of Medicine, Zamzam University College, Khartoum 11113, Sudan

**Keywords:** peptide receptor radionuclide therapy, somatostatin analogs, cytotoxic chemotherapy, pancreatic neuroendocrine tumor, non-surgical treatments

## Abstract

**Background:** Pancreatic neuroendocrine tumor (pNET) is a rare and complex disease that requires careful management and treatment. Currently, a range of treatments, including surgery, somatostatin analogs (SSA), peptide receptor radionuclide therapy (PRRT), targeted drugs, cytotoxic chemotherapy, and immunotherapy, exist for pNETs. However, determining the optimal treatment strategies remains challenging. **Aim:** To evaluate the efficacy and safety of non-surgical therapies, such as somatostatin analogs (SSA), peptide receptor radionuclide therapy (PRRT), targeted drugs, cytotoxic chemotherapy, and immunotherapy in treating pNETs. **Methods:** We systematically searched PubMed, Embase, the Cochrane Library, and Web of Science databases for relevant studies published from inception until August 2025. Randomized clinical trials (RCTs), non-randomized clinical trials, and prospective studies were included in this meta-analysis if they evaluated the efficacy and safety of any treatment of interest in patients with pNETs. **Results:** Thirty-three studies involving 2374 pNET patients were analyzed. Targeted therapies showed modest objective response rates (ORRs) but high disease control rates (DCRs): everolimus (ORR 7%, 95% CI: 3–10%; DCR 81%, 95% CI: 75–87%), sunitinib (ORR 12%, 95% CI: 5–19%; DCR 79%, 95% CI: 70–88%), surufatinib (ORR 19%, 95% CI: 12–27%; DCR 81%, 95% CI: 73–89%). Cytotoxic chemotherapy demonstrated higher ORRs: dacarbazine-based (32%, 95% CI: 21–43%), streptozocin-based (40%, 95% CI: 25–54%), temozolomide-based (42%, 95% CI: 29–55%). PRRT showed varying efficacy: ^177^Lu-DOTATATE (ORR 36%, 95% CI: 27–44%; DCR 84%, 95% CI: 76–92%), ^90^Y-DOTATOC (ORR 27%, 95% CI: 18–36%; DCR 73%, 95% CI: 63–83%). SSAs had low ORRs but high DCRs: lanreotide (ORR 0%, DCR 67%, 95% CI: 57–77%), octreotide (ORR 23%, 95% CI: 15–31%; DCR 75%, 95% CI: 66–84%). Immunotherapy with pembrolizumab showed limited efficacy (ORR 7%, 95% CI: 0–14%). Treatment-related adverse events were common across therapies, with specific toxicity profiles for each modality. **Conclusions:** Cytotoxic chemotherapy offers better response rates than other treatment modalities. However, toxicity management is crucial. PRRT also shows robust antitumor activity and disease control, while SSAs and targeted therapies are effective treatment options for disease stabilization. Immunotherapy demonstrated limited antitumor activity, and further research is needed to establish its role in pNET treatment.

## 1. Introduction

Pancreatic neuroendocrine tumors (pNETs) represent a rare oncological entity originating from the hormone-secreting cells of the pancreas. The annual incidence rate of these tumors is approximately 0.2 to 0.4 per 100,000 individuals; however, due to their relatively indolent progression, the actual incidence may be significantly higher [1,2,3]. At the time of diagnosis, 65% of patients typically present with metastatic or unresectable disease, which is associated with a five-year survival rate of approximately 30 to 40% [4]. Clinically, pNETs are classified as either functional or non-functional. Non-functional pNETs, which constitute 90% of cases, are characterized by the absence of a distinct hormonal hypersecretion syndrome [5,6]. These tumors often produce non-specific symptoms due to mass effects and are generally diagnosed in the fourth or fifth decade of life, frequently after metastasis to the liver has occurred. Conversely, functional pNETs, accounting for 10% of cases, manifest symptoms contingent upon the specific hormone being overproduced [7,8]. The most prevalent functional pNETs include insulinomas, gastrinomas, VIPomas, glucagonomas, and somatostatinomas. Over the past two decades, there has been a paradigm shift in the therapeutic approach to pNETs. Historically, treatment has relied on somatostatin analogues (SSAs), which remain a cornerstone therapy due to their effectiveness in managing hormone-related symptoms and inhibiting tumor growth. Recently, additional therapeutic modalities have emerged, including peptide receptor radionuclide therapy (PRRT), molecularly targeted agents such as everolimus and sunitinib, cytotoxic chemotherapy regimens, and immune checkpoint inhibitors. Nevertheless, the determination of optimal treatment strategies remains challenging, and ensuring precision in decision-making based on predictive models is highly complex [9,10].

Furthermore, the introduction of promising therapies in recent years has added another layer of complexity to treatment planning. The heterogeneity in study designs, patient selection, response criteria, and prior treatment exposure also limits the interpretation of individual trials and hamper evidence-based treatment selection. Consequently, the present meta-analysis was conducted to address the gaps by assessing the efficacy and safety of SSAs, PRRT, targeted therapies, cytotoxic chemotherapy, and immunotherapy, with the aim of informing treatment decisions for pNETs.

## 2. Methods

### 2.1. Eligibility Criteria

Studies were incorporated into the present meta-analysis if they conformed to the Population/Patients, Intervention, Comparison, Outcomes and Study design (PICOS) criteria delineated in Table 1. Additionally, inclusion was limited to studies published in English, those enrolling a minimum of 10 participants with pNETs, and those involving patients with NETs of any etiology, provided they separately reported the efficacy and/or safety outcomes for patients with pNETs.

### 2.2. Literature Search and Information Sources

A comprehensive search for pertinent studies published from inception until August 2025 was executed across the PubMed, Embase, Cochrane Library, and Web of Science databases. We adhered to the guidelines set by the Preferred Reporting Items for Systematic Reviews and Meta-Analyses (PRISMA). The search strategy incorporated terms such as pancreatic neuroendocrine tumors, surgery, chemotherapy, targeted therapies, somatostatin analogs, immunotherapy, and peptide receptor radionuclide therapy, utilizing the Boolean operators ‘AND’ and ‘OR.’ This electronic search was further augmented by manually reviewing the reference lists of included studies, grey literature (dissertations and theses), and previously published systematic reviews. The complete search terms employed in each electronic database are detailed in Appendix A. This systematic review and meta-analysis was registered prospectively in the PROSPERO database (CRD420251130658).

### 2.3. Data Extraction and Data Items

Two reviewers utilized a pre-established Excel spreadsheet to extract the data necessary for this meta-analysis. The information collected from each study encompassed the primary author’s name, year of publication, study design, patient characteristics (including the number of patients with pNETs, sex distribution, prior treatment, and characteristics of pNETs), interventions (dose and duration), and reported outcomes. The primary efficacy outcome was the objective response rate (ORR), defined as the best response rate (complete response (CR) + partial response (PR)), while the secondary efficacy outcome was the disease control rate (CR + PR + stable disease (SD)).

### 2.4. Quality Appraisal

The present study incorporated both randomized and non-randomized studies, necessitating the use of two separate quality appraisal tools for assessment. The Cochrane Risk of Bias tool (RoB-2) was employed to evaluate bias in randomized controlled trials (RCTs), while the ROBINS-I tool was utilized to assess bias in non-randomized studies. The RoB-2 tool facilitated the grading of RCTs as having low, high, or unclear bias across seven domains: random sequence generation, allocation concealment, blinding of participants and personnel, blinding of outcome assessment, incomplete outcome data, selective reporting, and other bias. Similarly, non-randomized studies were graded as having low, high, or unclear bias across seven domains, including confounding bias, classification of interventions, selection of participants, missing data, outcome measurement, and selection of reported results.

### 2.5. Data Synthesis

All statistical analyses were conducted utilizing STATA 18 software (Stata Corp, College Station, TX, USA). Data pertaining to ORR and DCR were aggregated in a one-arm meta-analysis, with the overall effect size determined using the untransformed (raw) proportion. To address the anticipated heterogeneity, all results were synthesized using the DerSimonian-Laird random effects model. Interstudy heterogeneity was assessed using the I^2^ statistic, where index values exceeding 50% indicated significant heterogeneity [11,12]. Additionally, subgroup analyses were performed based on the various therapeutic drugs employed in the treatment of pNET patients.

## 3. Results

### 3.1. Search Results

**Figure 1:**
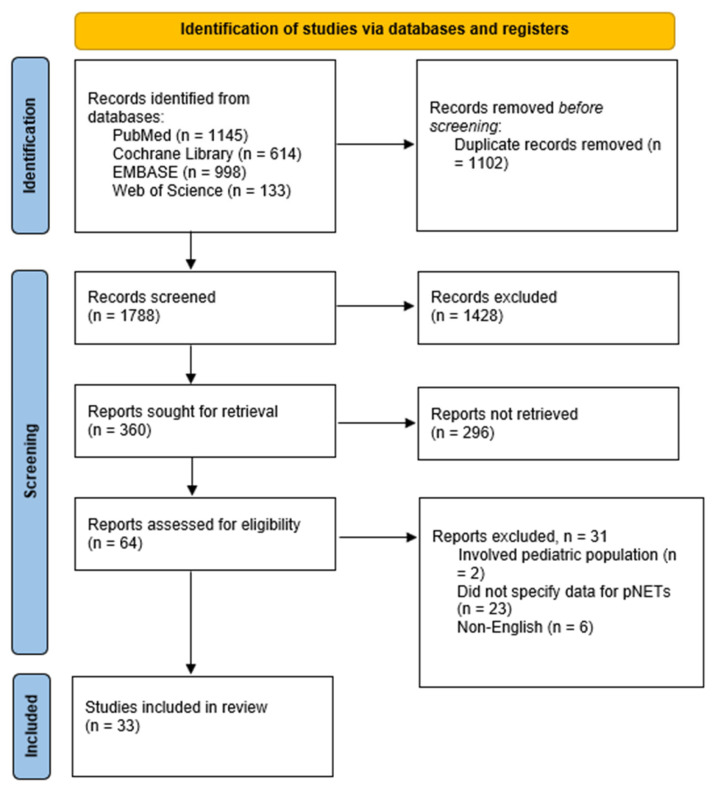
PRISMA flow diagram for study selection.

The electronic search yielded 2890 potential studies. These studies underwent a screening process for duplicates, resulting in the exclusion of 1102 studies. The titles and abstracts of the remaining studies were examined, and only 360 met the criteria for further consideration. Of these 360 studies, 296 were excluded due to their design as retrospective studies, case series, conference abstracts, meta-analyses, and narrative reviews. Ultimately, 33 studies were included, while the remaining 31 were excluded for the following reasons: six were non-English, 23 did not specify the outcomes of patients with pNETs separately, and two involved pediatric patients (Figure 1).

### 3.2. Characteristics of Included Studies

The 33 studies [13,14,15,16,17,18,19,20,21,22,23,24,25,26,27,28,29,30,31,32,33,34,35,36,37,38,39,40,41,42,43,44,45] included in this analysis enrolled a total of 2374 patients diagnosed with pancreatic neuroendocrine tumors (pNETs). Of these studies, eight evaluated the efficacy and safety of targeted therapies, specifically Sunitinib (n = 2) [15,16], Everolimus (n = 4) [13,17,18,20], Surufatinib (n = 1) [14], and Palbociclib (n = 1) [19]. Twelve studies focused on cytotoxic chemotherapy, with Dacarbazine-based chemotherapy (n = 2) [21,28], streptozocin-based chemotherapy (n = 5) [22,23,24,26,27], and temozolomide-based chemotherapy (n = 5) [28,29,30,31,32] being reported. Eight studies investigated peptide receptor radionuclide therapy (PRRT), including 90Y-DOTATATE (n = 1) [36], 90Y-DOTATOC (n = 2) [33], 177Lu-DOTATATE (n = 4) [34,38,39,40], and a combination of 90Y-DOTATOC and 177Lu-DOTATATE (n = 1) [35]. Three studies reported on somatostatin analogs (SSAs), specifically Lanreotide (n = 2) [41,42] and Octreotide (n = 1) [43]. Additionally, two studies [44,45] examined immunotherapy, specifically Pembrolizumab.

#### 3.2.1. Targeted Therapies

**Figure 2:**
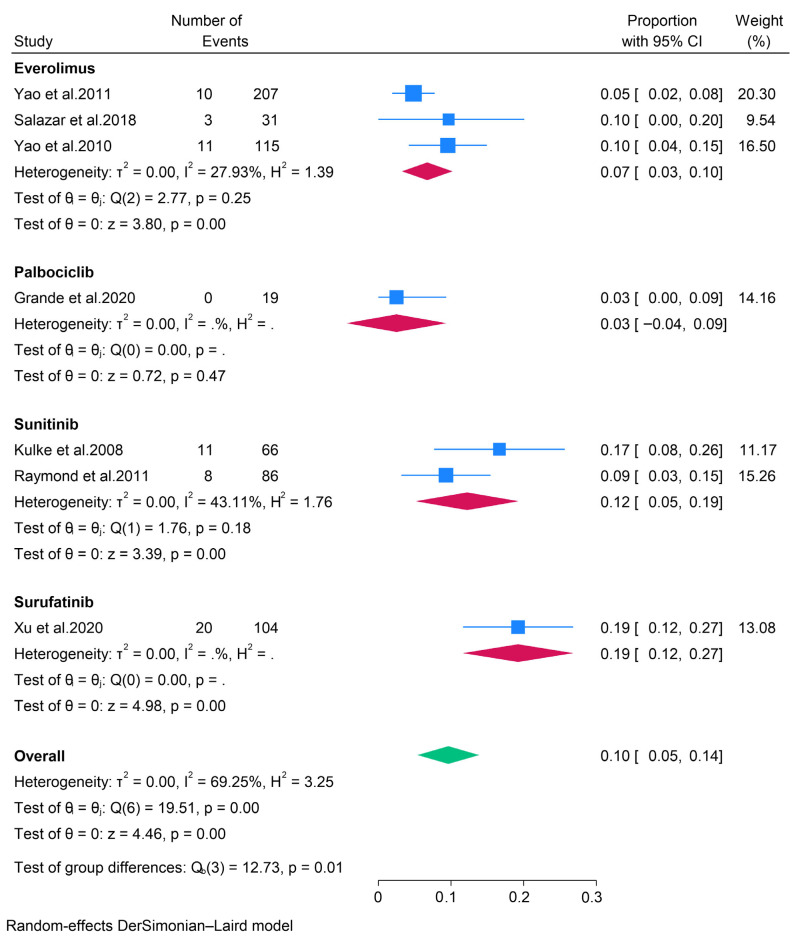
Forest plot showing the pooled ORR for patients treated with targeted therapies, i.e., everolimus, palbociclib, sunitinib, and surufatinib [13,14,15,16,17,18,19,20].

**Figure 3:**
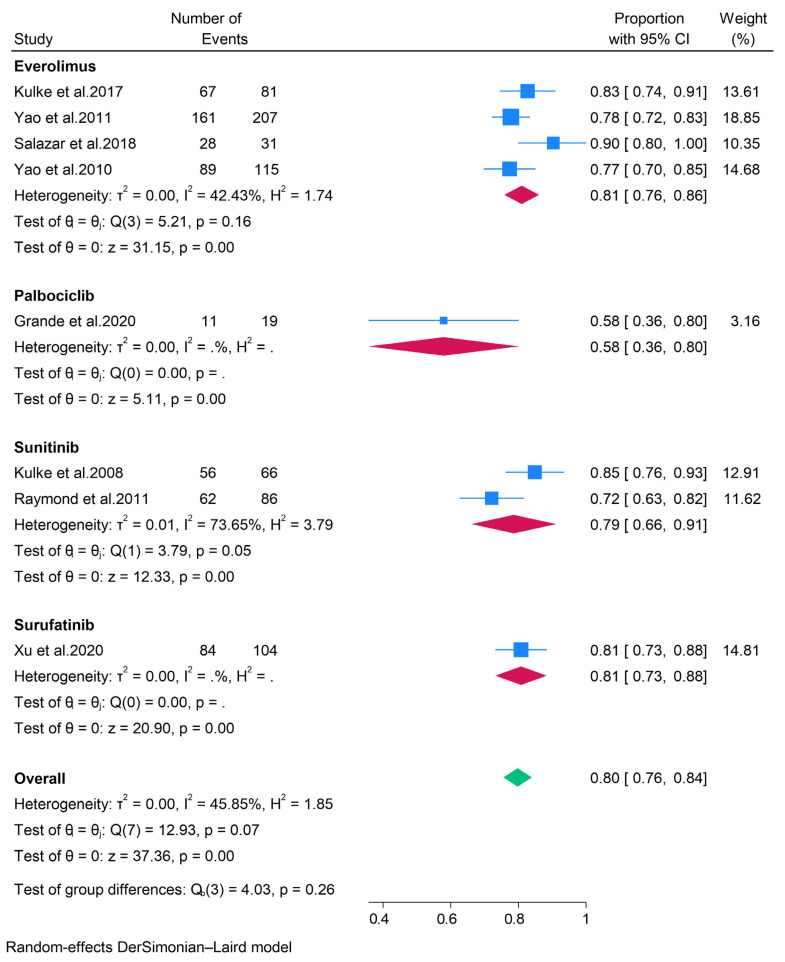
Forest plot showing the pooled DCR for patients treated with targeted therapies, i.e., everolimus, palbociclib, sunitinib, and surufatinib [13,14,15,16,17,18,19,20].

A subgroup analysis of various targeted therapies revealed that the objective response rates (ORR) for patients treated with everolimus, palbociclib, sunitinib, and surufatinib were 7% (95% CI: 3–10%), 3% (95% CI: 0–9%), 12% (95% CI: 5–19%), and 19% (95% CI: 12–27%), respectively (Figure 2). Additionally, the analysis indicated pooled disease control rates (DCR) of 81%, 58%, 79%, and 81% for patients receiving everolimus, palbociclib, sunitinib, and surufatinib, respectively (Figure 3). Concerning the safety profile of targeted therapies, data were pooled exclusively for patients treated with everolimus. The pooled data from the evaluable studies identified abdominal pain, diarrhea, rash, and stomatitis as the most prevalent adverse events, occurring in 30% or more of patients. Similarly, a multinational randomized controlled trial (RCT) involving patients with pancreatic neuroendocrine tumors (pNETs) treated with sunitinib reported diarrhea, nausea, asthenia, vomiting, and fatigue as the most common adverse events [16]. Furthermore, a study involving 21 patients with metastatic grade 1 and 2 pNETs found that the most frequent adverse events in patients treated with palbociclib were asthenia, neutropenia, diarrhea, and nausea [19].

#### 3.2.2. Cytotoxic Chemotherapy

**Figure 4:**
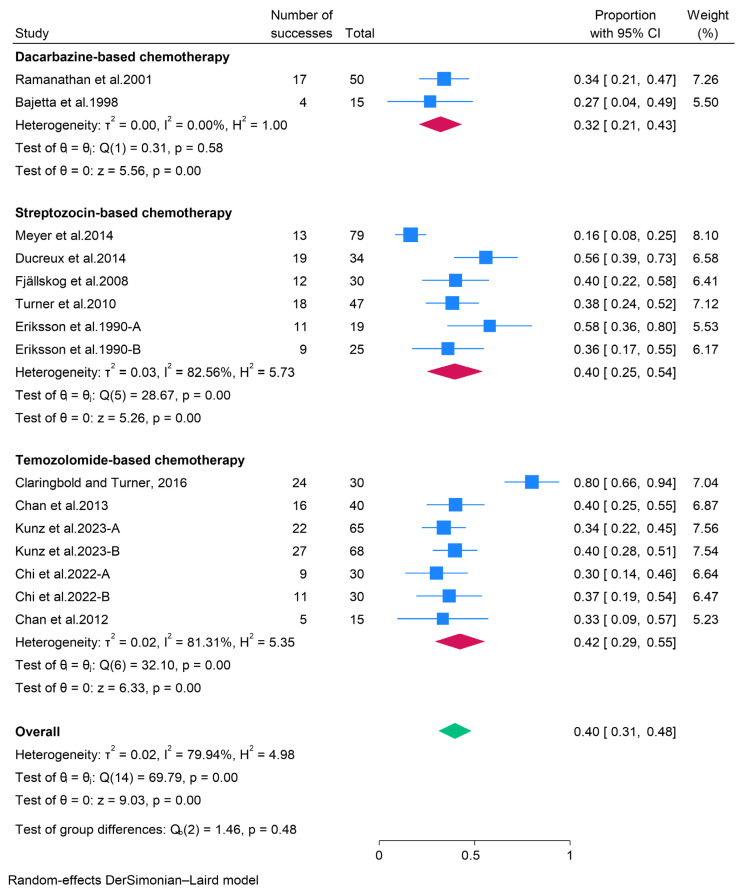
Forest plot showing the pooled ORR for patients treated with cytotoxic chemotherapy, i.e., dacarbazine-, streptozocin-, and temozolomide-based therapies [21,22,23,24,25,26,27,28,29,30,31].

**Figure 5:**
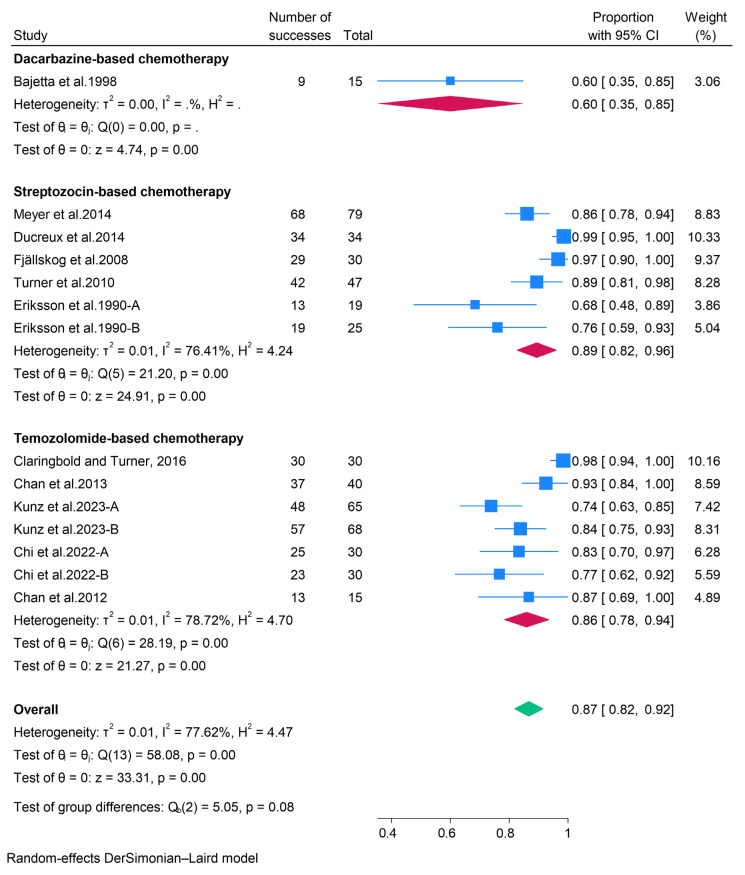
Forest plot showing the pooled DCR for patients treated with cytotoxic chemotherapy, i.e., dacarbazine-, streptozocin-, and temozolomide-based therapies [21,22,23,24,25,26,27,28,29,30,31].

In the present study, chemotherapy for pancreatic neuroendocrine tumors (pNETs) was categorized into dacarbazine, streptozocin, and temozolomide-based regimens. For patients receiving dacarbazine-based therapy, the pooled objective response rate (ORR) and disease control rate (DCR) were 32% (95% CI: 21–43%) and 60% (95% CI: 35–85%), respectively (Figure 4). The pooled data further indicated that pNETs treated with streptozocin-based therapy exhibited favorable response rates, with an ORR of 40% (95% CI: 25–54%) and a DCR of 89% (95% CI: 82–96%). Additionally, temozolomide-based therapies demonstrated a pooled ORR of 42% (95% CI: 29–55%) and a DCR of 86% (95% CI: 78–94%) (Figure 5). Among patients treated with temozolomide-based therapies, the most prevalent adverse events included anemia (49.2%), constipation (35.3%), diarrhea (41.9%), fatigue (69.7%), nausea (70.5%), decreased neutrophils (38%), oral mucositis (35.6%), decreased platelets (52.9%), and vomiting (38.4%). An open-label phase II trial (BETTER) involving 34 patients with progressive, metastatic, well-differentiated pNETs also reported that adverse events occurred in all patients treated with streptozocin-based therapy, with the most common adverse events being nausea (71%), asthenia (68%), and proteinuria (65%) [23].

#### 3.2.3. Peptide Receptor Radionuclide Therapy (PRRT)

**Figure 6:**
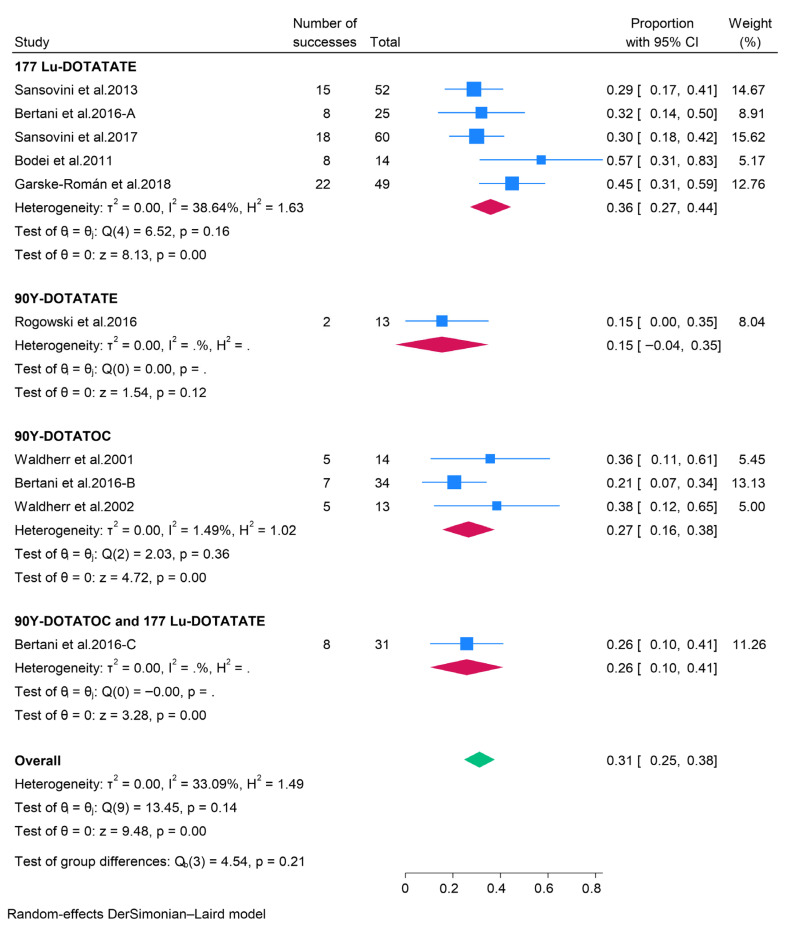
Forest plot showing the pooled ORR for patients treated with PRRT, i.e., 177Lu-DOTATATE, 90Y-DOTATATE, 90Y-DOTATOC, and combination of 177Lu-DOTATATE and 90Y-DOTATOC [33,34,35,36,37,38,39].

**Figure 7:**
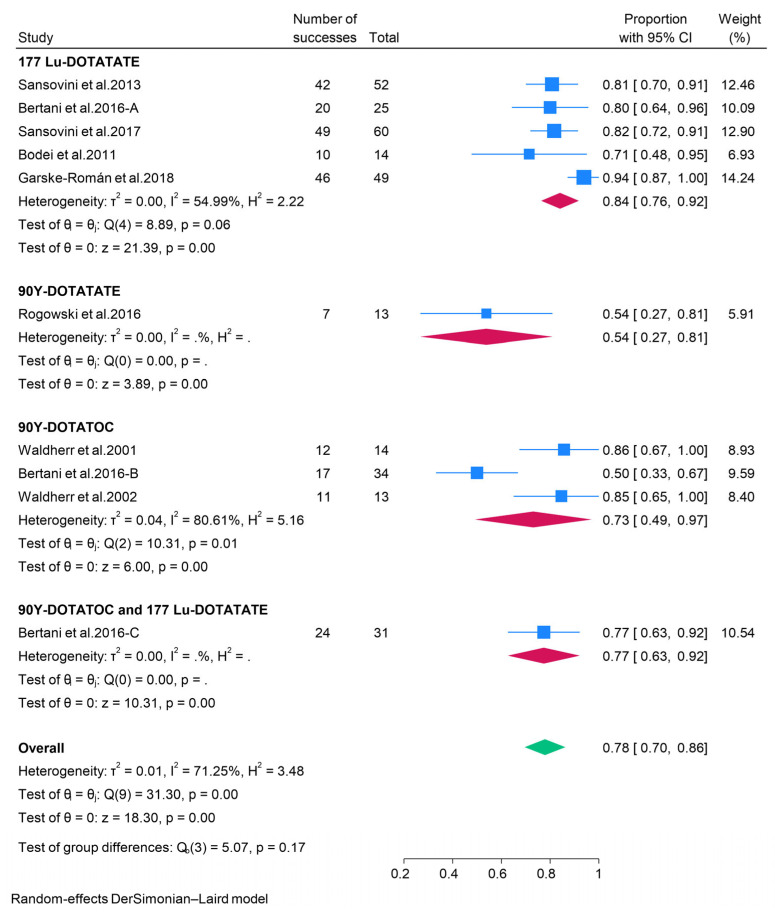
Forest plot showing the pooled DCR for patients treated with PRRT, i.e., 177Lu-DOTATATE, 90Y-DOTATATE, 90Y-DOTATOC, and combination of 177Lu-DOTATATE and 90Y-DOTATOC [33,34,35,36,37,38,39].

The effectiveness of 177Lu-DOTATATE peptide receptor radionuclide therapy (PRRT) in the treatment of pancreatic neuroendocrine tumors (pNETs) was documented in five studies. The aggregated data from these studies indicated that 36% (95% CI: 27–44%) of patients achieved an objective response rate (ORR), while the pooled disease control rate (DCR) was 84% (95% CI: 76–92%). Tumor response was also assessed in pNET patients undergoing treatment with 90Y-DOTATOC or 90Y-DOTATATE PRRT. The pooled ORR for 90Y-DOTATOC PRRT was 27%, with a DCR of 73%, whereas 90Y-DOTATATE PRRT exhibited a pooled ORR of 15% and a DCR of 54%. Furthermore, data from a single study indicated that patients treated with both 177Lu-DOTATATE and 90Y-DOTATOC achieved an ORR and DCR of 26% and 77%, respectively (Figure 6 and Figure 7). Due to the limited data on the safety of PRRT in patients with pNETs, a meta-analysis could not be performed. Consequently, safety outcomes were synthesized narratively. A prospective phase II clinical trial involving 63 patients with unresectable or metastatic pNETs treated with 177Lu-DOTATATE PRRT reported no significant adverse events necessitating the cessation of treatment. However, the most common minor adverse events included nausea, asthenia, and mild alopecia [34].

#### 3.2.4. Somatostatin Analogues (SSAs)

**Figure 8:**
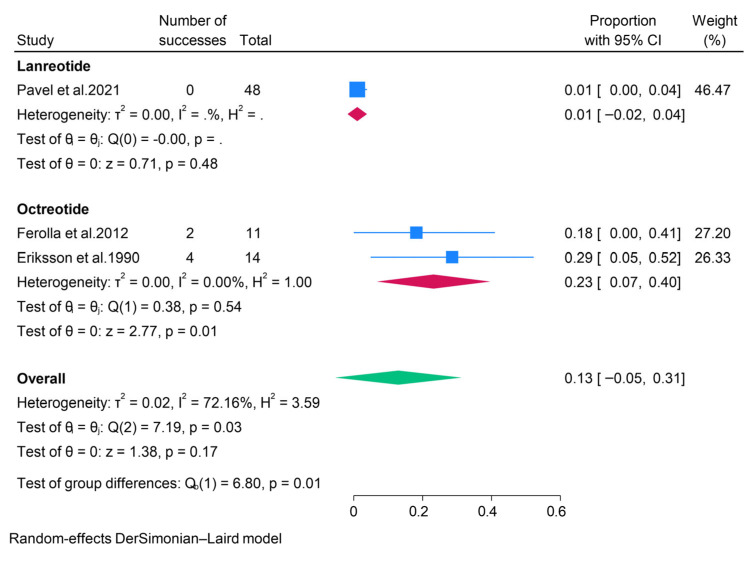
Forest plot showing the pooled ORR for patients treated with SSAs, i.e., lanreotide and octreotide [27,41,43].

**Figure 9:**
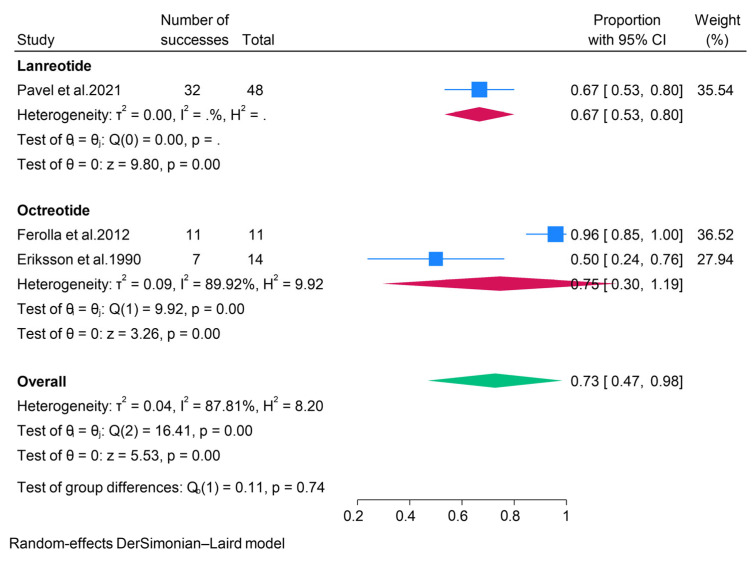
Forest plot showing the pooled DCR for patients treated with SSAs, i.e., lanreotide and octreotide [27,41,43].

The efficacy of somatostatin analogues (SSAs) was reported in three studies. A subgroup analysis according to the different SSAs revealed that ORR and DCR for pNET patients treated with Lanreotide were 0% and 67%, respectively. Moreover, we found a pooled ORR of 23% and DCR of 75% for pNETs treated with Octreotide (Figure 8 and Figure 9). None of the studies reporting the use of Octreotide assessed its safety in treating pNETs. However, the CLARINET FORTE study evaluated the safety of Lanreotide in pNET patients and found that treatment-related adverse events occurred in about a third of patients (37.5%), with the most common events being abdominal pain, diarrhea, and flatulence [41].

#### 3.2.5. Immunotherapy

**Figure 10:**
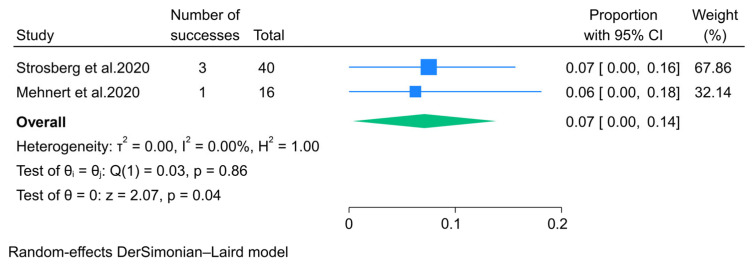
Forest plot showing the pooled ORR for patients treated with immunotherapy, specifically pembrolizumab [44,45].

The antitumor efficacy of immunotherapy, specifically pembrolizumab, has been documented in only two non-randomized clinical trials. The aggregated data from these trials indicated an overall response rate (ORR) of 7% (95% CI: 0–14%) (Figure 10). Additionally, findings from one of the trials demonstrated that 15 out of 16 patients with pancreatic neuroendocrine tumors (pNETs) experienced at least one adverse event, with diarrhea and fatigue being the most frequently reported treatment-related adverse events. Importantly, none of the patients with pNETs discontinued pembrolizumab due to treatment-related adverse events [45].

### 3.3. Meta-Regression Analysis

Table 2 shows a summary of the meta-regression analysis results. For Streptozocin-based therapy, the sample size and study design significantly influenced ORR, while the line of treatment impacted the DCR outcomes. For temozolomide-based therapy, the line of treatment was a significant source of heterogeneity for both ORR and DCR. Among the PRRT therapies, variations in sample size and response assessment criteria influenced the ORR for 177Lu-DOTATATE, while the response assessment criteria contributed to significant heterogeneity in the DCR for 90Y-DOTATOC.

### 3.4. Risk of Bias Outcomes

**Figure 11:**
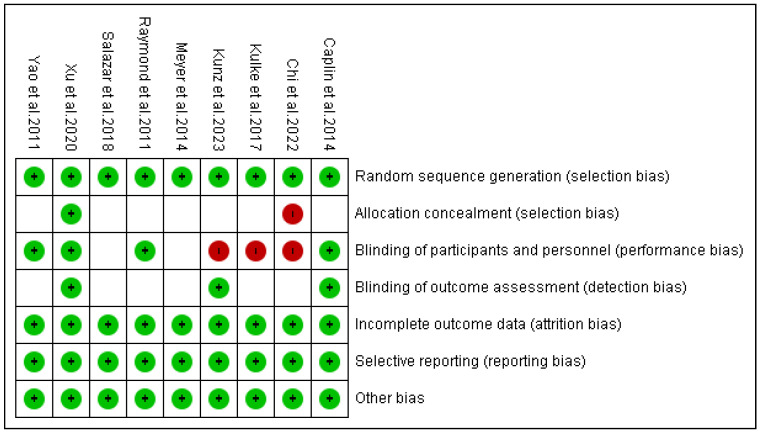
Summary of risk of bias using the RoB-2 tool [13,14,15,16,17,18,19,22,42].

**Figure 12:**
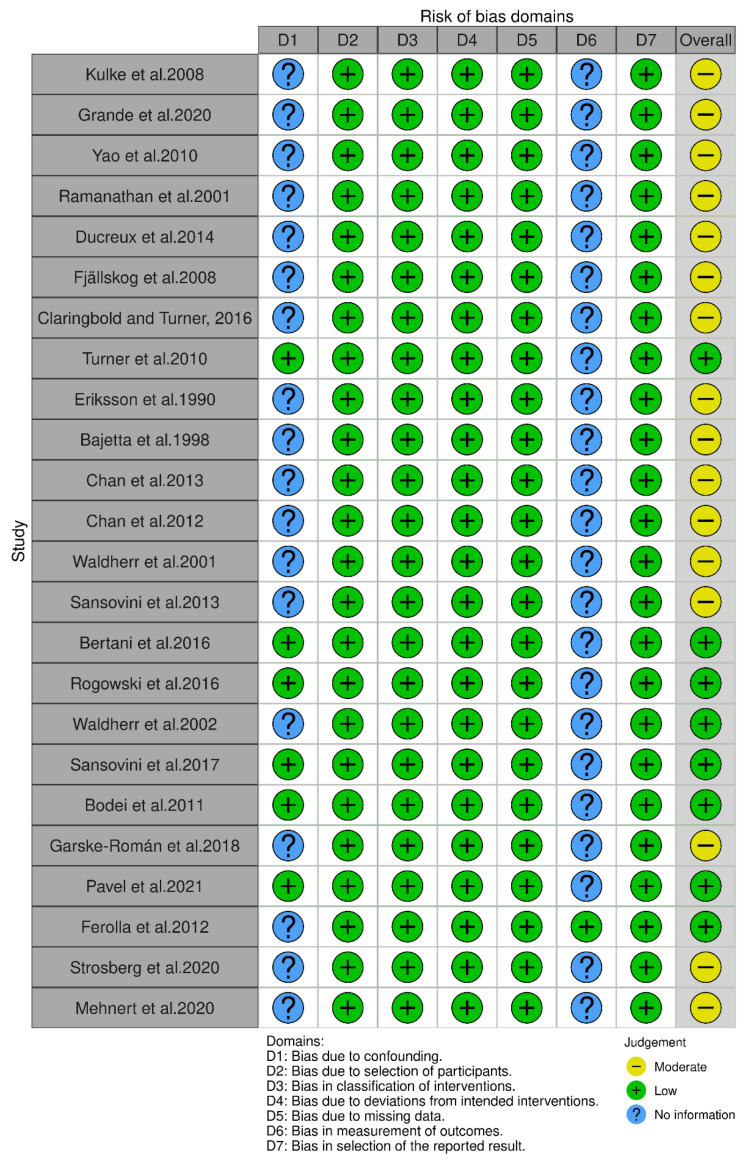
Summary of risk of bias using the ROBINS-I tool [15,19,20,21,23,24,25,26,27,28,29,34,35,36,37,38,39,40,41,43,44,45].

Figure 11 and Figure 12 present a summary of the risk of bias results. According to the RoB-2 tool, one RCT exhibited a high risk of selection bias due to the lack of concealment of drug allocation from patients. Furthermore, three RCTs showed performance bias as they were open-label and did not implement blinding for participants and staff.

## 4. Discussion

The present meta-analysis has consolidated the extant evidence regarding the efficacy and safety of therapeutic interventions for patients with pancreatic neuroendocrine tumors (pNETs), encompassing targeted therapies, cytotoxic chemotherapy, peptide receptor radionuclide therapy (PRRT), somatostatin analogs (SSAs), and immunotherapy. Our findings illuminate the diverse yet complementary roles of these modalities in the management of pNETs, underscoring the necessity for personalized treatment strategies. Currently, the European Medicines Agency and the Food and Drug Administration (FDA) have sanctioned two targeted therapies for pNET treatment: the tyrosine kinase inhibitor sunitinib and the mTOR inhibitor everolimus [46,47]. Nonetheless, other targeted therapies, such as Surufatinib and Palbociclib, have also been subject to investigation [14,19]. Our results indicate that the overall objective response rates (ORR) in pNET patients treated with sunitinib, everolimus, surufatinib, and palbociclib were 12%, 7%, 19%, and 3%, respectively. Despite these modest ORRs, the disease control rates (DCR) achieved by patients receiving these therapies are relatively high (79%, 81%, 58%, and 81% for sunitinib, everolimus, palbociclib, and surufatinib, respectively), suggesting that targeted therapies are valuable for disease management, even if they do not result in significant tumor reduction. Furthermore, evidence indicates that abdominal pain, diarrhea, rash, and stomatitis are common adverse effects of everolimus. In contrast, diarrhea, nausea, asthenia, vomiting, and fatigue are predominant with sunitinib, while asthenia, neutropenia, diarrhea, and nausea are more frequent with surufatinib. Despite these adverse effects, the rates of treatment discontinuation are modest, implying that targeted therapies are generally tolerable. Cytotoxic chemotherapy has been a longstanding therapeutic option for patients with differentiated pNETs, particularly temozolomide-based and streptozocin-based combinations. Our analysis reveals that cytotoxic chemotherapy yields superior ORRs compared to other approaches (32%, 40%, and 42% for dacarbazine-based, streptozocin-based, and temozolomide-based therapies, respectively), highlighting its clinical desirability for tumor reduction. Additionally, chemotherapy demonstrated high DCRs, supporting its efficacy in disease control. Nonetheless, patients undergoing chemotherapy exhibit high incidences of adverse events. Indeed, the BETTER trial involving 34 patients with progressive well-differentiated pNETs reported that all participants experienced at least one adverse event following treatment with Bevacizumab plus 5-FU and streptozocin [23]. A phase II trial of patients with advanced pancreatic islet carcinoma also found that 30% of patients treated with dacarbazine experienced grade 3–4 toxicities [21]. Therefore, although chemotherapy offers enhanced tumor activity, effective management of toxicity is crucial.

Somatostatin analogues (SSAs) are recognized as first-line therapeutic agents for pancreatic neuroendocrine tumors (pNETs) that overexpress somatostatin receptors [48,49]. Prominent SSAs include octreotide long-acting release (LAR) and lanreotide autogel, which primarily target SSTR-2 (expressed in approximately 80% of pNETs [50]) and SSTR-5. In the current study, both lanreotide and octreotide exhibited modest objective response rates (ORRs) but high disease control rates (DCRs), suggesting that the principal therapeutic advantage of SSAs lies in tumor stabilization. Furthermore, SSAs have demonstrated low incidences of toxicities, with the CLARINET FORTE study indicating that adverse events occurred in approximately one-third of patients treated with lanreotide [41]. This low toxicity, combined with the disease-stabilization effect, supports the continued use of SSAs as first-line treatment for pNETs overexpressing somatostatin receptors. In comparison to SSAs, peptide receptor radionuclide therapy (PRRT) exhibited superior ORRs, underscoring its efficacy in tumor reduction among patients with advanced receptor-positive pNETs. Our findings also revealed that PRRT has high DCRs, indicating its capacity to induce responses and maintain disease stabilization. These results align favorably with a previous meta-analysis, which reported that 177Lu-DOTATATE PRRT had a pooled ORR of 47% and DCR of 81% among patients with advanced pNETs [51]. Additionally, evidence suggests that PRRT has a favorable toxicity profile [34]. Therefore, PRRT may represent an effective and safe treatment option for well-differentiated pNETs that progress following SSAs. The present study also demonstrated that immunotherapy has a pooled ORR of 7%, indicating that immunotherapy with pembrolizumab may elicit responses in patients with pNETs. However, the response rates are relatively low, and a significant proportion of patients may experience treatment-related side effects. Indeed, Mehnert and colleagues reported that treatment-related adverse events occurred in 68.8% of pNET patients treated with pembrolizumab [45]. Given the high toxicity rate and modest ORR, immunotherapy remains an investigational treatment for pNETs, and further research is necessary to better understand the factors influencing the efficacy of immunotherapy.

### 4.1. Comparison with Existing Evidence

The high response rates observed in patients treated with cytotoxic chemotherapy are comparable to a previous meta-analysis assessing the efficacy of systemic therapies in patients with advanced well-differentiated pNETs [49]. According to that study, chemotherapy, specifically capecitabine/temozolomide, showed the best results, with the objective response (tumor shrinkage of ≥10%) ranging from 65% to 93%. These findings confirm the utility of cytotoxic chemotherapy as first-line therapy due to its improved tumor activity.

The pooled ORR and DCR for PRRT in this meta-analysis also compare favorably with prior review articles. For instance, a meta-analysis evaluating the therapeutic efficacy of 177Lu-DOTATATE PRRT in patients with advanced pNETs reported pooled ORR of 47% and DCR of 81% [49]. Another systematic review evaluating the use of 177Luteciuim- DOTA-conjugated peptides and 90Yetrium- DOTA- conjugated peptides reported that PRRT is an effective treatment option for patients with non-operable and/or metastatic pNETs, with complete responses ranging between 2 and 6% and partial response being observed in up to 60% of the cases [50]. For targeted therapies, the pooled efficacy of everolimus (ORR: 7%) closely aligns with the outcomes reported in a meta-analysis of 12 articles, which found a pooled ORR of 12% [51].

### 4.2. Limitations

The present meta-analysis is subject to several limitations. Firstly, considerable heterogeneity was observed across most of the pooled results. This heterogeneity is likely attributable to variations in sample size, response assessment criteria, study design, and prior treatments. Nonetheless, we mitigated the impact of this heterogeneity on the pooled outcomes by employing a random-effects model. Secondly, due to a lack of sufficient data on predictors of response, we were unable to conduct a meta-analysis in this context. Consequently, future prospective trials should aim to incorporate predictors of response to enhance understanding of which patients are likely to benefit from various therapies. Lastly, we included only studies published in English, which may have introduced selection bias into our analyses.

## 5. Conclusions

Our meta-analysis has established that cytotoxic chemotherapy yields higher response rates compared to other treatment modalities. Consequently, cytotoxic chemotherapy may be considered a first-line treatment, particularly for patients with pancreatic neuroendocrine tumors (pNETs) who exhibit symptoms due to tumor burden or for those where cytoreduction could provide a clinical benefit. Nonetheless, the management of toxicity is crucial during cytotoxic chemotherapy treatment. Peptide receptor radionuclide therapy (PRRT) also exhibits significant antitumor activity and high disease stabilization, suggesting that it may be an effective and safe treatment option for well-differentiated pNETs that progress following somatostatin analogs (SSAs). Additionally, SSAs and targeted therapies have demonstrated high disease control rates (DCR), underscoring their role in disease stabilization. Conversely, immunotherapy appears to have a limited role in tumor activity, thus remaining an investigational treatment for pNETs. Further research is necessary to elucidate the factors influencing its efficacy.

## Figures and Tables

**Table 1:** Inclusion and Exclusion Criteria.

Criterion	Included	Excluded
Population (P)	Adults (age ≥ 18 years) with pancreatic neuroendocrine tumors and without restriction on previous treatment.	Children (<18 years) or patients with non-pancreatic neuroendocrine tumors
Interventions (I)	Surgery, chemotherapy, targeted therapies, somatostatin analogs, immunotherapy, or peptide receptor radionuclide therapy.	Liver-directed therapies include chemoembolization, radioembolization, radiofrequency ablation, liver transplant, and arterial embolization.
Control (C)	Placebo, usual care, or no treatment	-
Outcomes (O)	Efficacy-related outcomes, such as objective response rate (ORR) and disease control rate (DCR) Safety outcomes, i.e., adverse events	-
Study Design (S)	Randomized controlled trials (RCTs), non-randomized clinical trials, or single-arm prospective studies.	Retrospective studies, case series, narrative reviews, conference/meeting abstracts, letters to the editor, case reports, meta-analyses, or commentaries.

**Table 2:** Meta-Regression Analysis investigating the sources of heterogeneity.

Treatment	Outcome	Covariate	Regression Coefficient	*p*-Value	Residual Heterogeneity Test
Streptozocin-based therapy	ORR	Sample size	−0.281	0.0002	Q_res: 4.99 *p* = 0.289
		Study design	−0.281	0.0002	Q_res: 4.99 *p* = 0.289
		Response assessment criteria	−0.085	0.577	Q_res: 22.80 *p* = 0.0001
		Line of treatment	−0.097	0.558	Q_res: 24.41 *p* = 0.0001
	DCR	Sample size	−0.041	0.649	Q_res: 15.98 *p* = 0.003
		Study design	−0.041	0.649	Q_res: 15.98 *p* = 0.003
		Response assessment criteria	0.073	0.392	Q_res: 20.41 *p* = 0.0004
		Line of treatment	0.205	0.01	Q_res: 10.49 *p* = 0.033
Temozolomide-based therapy	ORR	Sample size	−0.0799	0.599	Q_res: 24.48 *p* < 0.001
		Study design	−0.181	0.124	Q_res: 19.54 *p* = 0.002
		Line of treatment	−0.439	<0.0001	Q_res: 1.36 *p* = 0.929
	DCR	Sample size	−0.105	0.134	Q_res: 14.01 *p* = 0.016
		Study design	−0.162	<0.0001	Q_res: 5.31 *p* = 0.380
		Line of treatment	−0.150	0.016	Q_res: 8.63 *p* = 0.125
177Lu-DOTATATE	ORR	Sample size	−0.133	0.048	Q_res: 2.62 *p* = 0.454
		Response assessment criteria	0.133	0.048	Q_res: 2.62 *p* = 0.454
	DCR	Sample size	−0.058	0.425	Q_res: 5.20 *p* = 0.157
		Response assessment criteria	0.058	0.425	Q_res: 5.20 *p* = 0.157
90Y-DOTATOC	ORR	Response assessment criteria	−0.164	0.156	Q_res: 0.02 *p* = 0.883
	DCR	Response assessment criteria	−0.352	0.001	Q_res: 0.01 *p* = 0.936

## Data Availability

No new data were created or analyzed in this study. Data sharing is not applicable to this article.

## Abbreviations

The following abbreviations are used in this manuscript:

5-FU	5-Fluorouracil
CI	Confidence Interval
CR	Complete Response
DCR	Disease Control Rate
DOTATATE	DOTA-Tyr3-Octreotate
DOTATOC	DOTA-Tyr3-Octreotide
FDA	Food and Drug Administration
IV	Intravenous
LAR	Long-Acting Release
Lu	Lutetium
N/A	Not Applicable
NET	Neuroendocrine Tumor
NR	Not Reported
ORR	Objective Response Rate
pNET	Pancreatic Neuroendocrine Tumor
PICOS	Population, Intervention, Comparison, Outcomes, and Study Design
PR	Partial Response
PRRT	Peptide Receptor Radionuclide Therapy
RCT	Randomized Controlled Trial
RoB-2	Cochrane Risk of Bias tool version 2
ROBINS-I	Risk Of Bias In Non-randomized Studies-of Interventions
SD	Stable Disease
SSA	Somatostatin Analog
SSTR	Somatostatin Receptor
WHO	World Health Organization
Y	Yttrium

## Appendix A. Search Strategy

- PubMed

[All Fields] (Pancreatic neuroendocrine tumors OR neuroendocrine tumors OR gastroenteropancreatic neuroendocrine tumors OR insulinomas OR gastrinomas OR VIPomas OR glucagonomas OR somatostatinomas) AND (chemotherapy OR cytotoxic chemotherapy OR streptozocin OR temozolomide OR dacarbazine OR targeted therapies OR sunitinib OR everolimus OR surufatinib OR palbociclib OR somatostatin analogues OR immunotherapy OR immune checkpoint inhibitors) AND (Randomized clinical trial OR Prospective OR clinical trial)

[Title/Abstract] (Pancreatic neuroendocrine tumors OR neuroendocrine tumors OR gastroenteropancreatic neuroendocrine tumors OR insulinomas OR gastrinomas OR VIPomas OR glucagonomas OR somatostatinomas) AND (chemotherapy OR cytotoxic chemotherapy OR streptozocin OR temozolomide OR dacarbazine OR targeted therapies OR sunitinib OR everolimus OR surufatinib OR palbociclib OR somatostatin analogues OR immunotherapy OR immune checkpoint inhibitors) AND (Randomized clinical trial OR Prospective OR clinical trial)

- Embase

(Pancreatic neuroendocrine tumors OR neuroendocrine tumors OR gastroenteropancreatic neuroendocrine tumors OR insulinomas OR gastrinomas OR VIPomas OR glucagonomas OR somatostatinomas) AND (chemotherapy OR cytotoxic chemotherapy OR streptozocin OR temozolomide OR dacarbazine OR targeted therapies OR sunitinib OR everolimus OR surufatinib OR palbociclib OR somatostatin analogues OR immunotherapy OR immune checkpoint inhibitors) AND (Randomized clinical trial OR Prospective OR clinical trial)

- Cochrane Library

(Pancreatic neuroendocrine tumors OR neuroendocrine tumors OR gastroenteropancreatic neuroendocrine tumors OR insulinomas OR gastrinomas OR VIPomas OR glucagonomas OR somatostatinomas) AND (chemotherapy OR cytotoxic chemotherapy OR streptozocin OR temozolomide OR dacarbazine OR targeted therapies OR sunitinib OR everolimus OR surufatinib OR palbociclib OR somatostatin analogues OR immunotherapy OR immune checkpoint inhibitors) AND (Randomized clinical trial OR Prospective OR clinical trial)

- Web of Science

(Pancreatic neuroendocrine tumors OR neuroendocrine tumors OR gastroenteropancreatic neuroendocrine tumors OR insulinomas OR gastrinomas OR VIPomas OR glucagonomas OR somatostatinomas) AND (chemotherapy OR cytotoxic chemotherapy OR streptozocin OR temozolomide OR dacarbazine OR targeted therapies OR sunitinib OR everolimus OR surufatinib OR palbociclib OR somatostatin analogues OR immunotherapy OR immune checkpoint inhibitors) AND (Randomized clinical trial OR Prospective OR clinical trial)
